# Association of dietary inflammatory index with helicobacter pylori infection and mortality among US population

**DOI:** 10.1186/s12967-023-04398-8

**Published:** 2023-08-12

**Authors:** Yu-Jun Xiong, Lei-Lei Du, Yun-Lian Diao, Jun Wen, Xiang-Bin Meng, Jun Gao, Chun-Li Shao, Wen-Yao Wang, Xing-yun Zhu, Yi-Da Tang

**Affiliations:** 1 Department of Gastroenterology, Beijing Hospital, National Center of Gerontology, Institute of Geriatric Medicine, Chinese Academy of Medical Sciences, Beijing, People’s Republic of China 100370; 2 Department of Cardiology, Cardiovascular Center, Beijing Friendship Hospital, Capital Medical University, 95 Yongan Road, Beijing, 100050 China; 3 Department of Respiratory and Critical Care Medicine, Jiangxi Institute of Respiratory Disease, The First Affiliated Hospital of Nanchang University, Nanchang, China; 4 Department of Cardiology and Institute of Vascular Medicine, Peking University Third Hospital; Key Laboratory of Molecular Cardiovascular Science, Ministry of Education, No. 49 Huayuanbei Road, BeijingBeijing, 100191 China; 5 Department of Endocrinology, Beijing Jishuitan Hospital, No. 31, East Xinjiekou Street, Xicheng District, 100035 Beijing, People’s Republic of China

**Keywords:** H. pylori infection, DII, NHANES, Mortality

## Abstract

**Background:**

Limited research has been conducted on the potential relationship between the dietary inflammation index (DII) and mortality, particularly in individuals with Helicobacter pylori (H. pylori) infection. This study aimed to investigate the association between the DII and H. pylori infection, as well as their respective impacts on all-cause mortality in a cohort of individuals with or without H. pylori infection.

**Methods:**

Data from the 1999–2018 National Health and Nutrition Examination Survey (NHANES) were utilized for this study, with a final of 4370 participants included. Both univariable and multivariable-adjusted logistic regression analyses were employed to explore the relationship between H. pylori infection and pertinent covariates. Cox regression analysis, as well as restricted regression cubic spline analysis, were utilized to assess the association between DII and all-cause mortality among individuals with or without H. pylori infection.

**Results:**

The findings demonstrated a positive correlation between DII scores and H. pylori infection, even after adjusting for potential confounding factors. Moreover, higher DII scores were significantly associated with an elevated risk of mortality exclusively in individuals with H. pylori infection, while no such association was observed in the uninfected population. Additional analysis using restricted cubic spline modeling revealed a positive linear relationship between DII scores as a continuous variable and the adjusted risk of all-cause mortality specifically in H. pylori-infected patients.

**Conclusion:**

The results of this study indicated that DII was positively correlated with an increased risk of H. pylori infection and was associated with a heightened risk of all-cause mortality solely in individuals with H. pylori infection. Consequently, DII might serve as a useful tool for risk stratification in the H. pylori-infected population among U.S. adults. Further research is warranted to elucidate the underlying mechanisms and potential clinical implications of these findings.

## Introduction

Helicobacter pylori (H. pylori), a gram-negative bacterium, infects the human stomach and is strongly associated with various gastric diseases, including gastritis, peptic ulcer disease and gastric cancer [1]. Its prevalence is typically high in developing countries and certain regions, such as Africa and Latin America, with an estimated global prevalence of 50% [2]. H. pylori infection rates are similar between genders, with 42.7% in women and 46.3% in men [3]. Among the various risk factors, diet has emerged as an important factor influencing H. pylori infection [4, 5].

Prior evidence suggests that diet quality and inflammation may be linked to H. pylori infection. Unhealthy dietary patterns have been associated with a higher risk of H. pylori infection and an increased likelihood of developing H. pylori-related diseases [6]. The dietary inflammatory index (DII), a scoring system designed to assess the overall quality and composition of the diet in terms of its impact on inflammation in the body [7], has been widely used to investigate the association between diet-related inflammation and the risk of infection. Previous studies have demonstrated that a positive association between higher DII scores and an increased risk of infectious diseases, including gastrointestinal infections [8], which may be relevant to H. pylori infection. Conversely, adhering to an anti-inflammatory diet, characterized by high consumption of fruits, vegetables, whole grains, and healthy fats, has been linked to a reduced risk of infections [9]. Recent research has also indicated that the intake of nutrient antioxidants may decrease the risk of H. pylori infection [10].

In addition, several studies have also investigated the association between diet and mortality. In a prospective cohort study conducted on 3521 adults of normal BMI, diet of high DII scores was associated with an increased risk of cardiovascular disease (CVD) mortality [11]. Another study utilizing data from the National Health and Nutrition Examination Survey (NHANES) database found that dietary patterns with low DII scores were negatively associated with the risk of all-cause mortality in individuals over 60 years old [12]. Moreover, as classified by the World Health Organization (WHO), H. pylori is considered a Group 1 carcinogen for gastric cancer [13], leading to the development of precancerous lesions and increase the likelihood of gastric cancer, and therefore an increased risk of death.

Hence, this study aims to explore the relationship between the DII score and H. pylori infection, and investigate whether the associations between DII scores and all-cause mortality are mediated by H. pylori infection using the data in the NHANES database.

## Materials and methods

### Study design and participants

The NHANES project employs a sophisticated and intricate methodology, to select a representative sample of the U.S. population every two years. Its primary objective is to evaluate and assess the health and nutritional status of individuals in the United States [14]. To ensure ethical standards, the survey has received approval from The National Center for Health Statistics Institutional Review Board. Moreover, all participants have willingly provided written informed consent before their inclusion in the study. NHANES collects a wide range of data, including demographics, dietary patterns, medical examination results, laboratory findings, and responses to questionnaires [15].

**Fig. 1:**
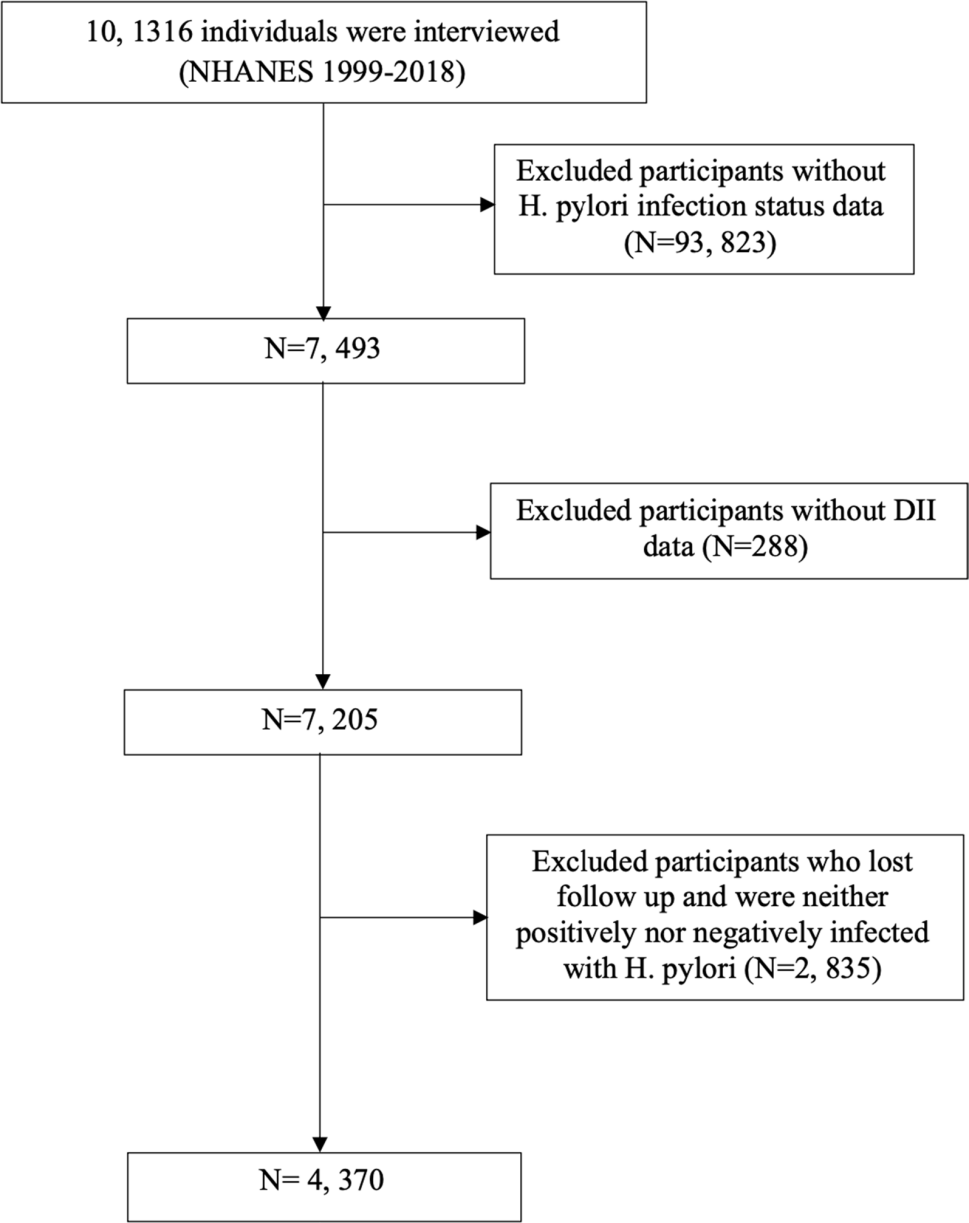
Flow chart for inclusion and exclusion of the study population

In the NHANES 1999–2018 cycle, a total of 101,136 individuals took part in the study. After excluding subjects without information on H. pylori infection status or dietary recall, as well as those lost follow up, the remaining sample was used for analysis (Fig. 1).

### Dietary inflammatory index

The dietary inflammatory index (DII) is a comprehensive scoring system that was developed to assess the potential inflammatory effects of dietary intake. In this study, the calculation of the DII involved the inclusion of 26 nutrients. These nutrients encompassed alcohol, vitamin B12/B6, β-carotene, caffeine, carbohydrate, cholesterol, total fat, fiber, folic acid, iron, magnesium, zinc, selenium, monounsaturated fatty acids, niacin, n-3 fatty acids, n-6 fatty acids, protein, polyunsaturated fatty acids, riboflavin, saturated fat, thiamin, and vitamins A/C/E. Importantly, it should be noted that even if fewer than 30 nutrients are utilized, the DII can still be computed with reliable accuracy [7]. In the scoring system, foods and nutrients with anti-inflammatory properties receive negative scores, while pro-inflammatory substances receive positive scores [16]. These individual scores are subsequently summed to generate a comprehensive DII score representing the overall inflammatory potential of an individual's diet.

### Helicobacter pylori status

NHANES utilized an enzyme-linked immunoassay (ELISA) to evaluate H. pylori exposure by detecting IgG antibodies [17]. The NHANES datasets did not include the specific IgG results. The ELISA method demonstrated comparable sensitivity, specificity, and reproducibility to other antibody serological tests such as immunofluorescence, complement fixation, hemagglutination, and radioimmunoassays [18, 19]. To classify participants as H. pylori seropositive (optical density (OD) value ≥ 1.1) or seronegative (OD value < 0.9), standard ELISA cut-offs were employed [20]. Equivocal values (0.9–1.1) were excluded from the analysis to ensure accurate statistical outcomes in this study.

### Follow up and endpoint

The participants' mortality status and cause of death were determined by linking their records to the National Death Index public access files, with a repetition rate of less than 10%, until December 31, 2019 (https://www.cdc.gov/nchs/data-linkage/mortality.htm). The median follow-up time was 235 (interquartile range 184, 243) months in H. pylori positively infected participants while in H. pylori negatively infected patients it was 236 (229, 242) months.

### Covariate

The survey questionnaire obtained information on several factors from participants, including age, gender, race, poverty, education, smoking habits, alcohol drinking, diabetes mellitus, hypertension status, cardiovascular disease, stroke, heart attack, frailty score, triglyceride-glucose (TyG) index and DII. Hypertension was defined as a systolic blood pressure (SBP) ≥ 140 mmHg and/or diastolic blood pressure (DBP) ≥ 90 mmHg, or as having received antihypertensive treatment [21]. Smoking status was categorized into three groups: non-smokers, former smokers, and current smokers. Non-smokers were individuals who had either never smoked or had smoked fewer than 100 cigarettes in their lifetime. Former smokers were defined as those who had previously smoked at least 100 cigarettes but were not currently smoking. Current smokers were participants who had smoked at least 100 cigarettes in their lifetime and reported consuming a nonzero number of cigarettes per day within the past 30 days [22]. Alcohol drinking status was classified into four distinct categories, reflecting their alcohol consumption patterns. Never drinkers were individuals who reported consuming less than 12 drinks in their lifetime. Former drinkers were defined as participants who had consumed more than 12 drinks in their lifetime but had not consumed any alcohol in the past year. Current drinkers were further classified as mild, moderate, or heavy drinkers. Heavy current drinkers were defined as women who consumed three or more drinks per day or men who consumed four or more drinks per day, with at least five or more binge drinking episodes per month. Moderate drinkers were defined as women who consumed two or more drinks per day or men who consumed three or more drinks per day [23]. Moreover, the educational level was categorized into 4 groups: College or above, high school or equivalent, less than high school, and more than high school.

### Statistical analysis

Baseline characteristics of participants were summarized and compared by H. pylori infected and uninfected patients. Continuous variables were expressed as mean (± SD) and compared by *t* test or Wilcoxon rank-sum test, depending on the result of Kolmogorov–Smirnov normality test. Categorical variables were presented as frequency (percentage) and compared by Chi-square test.

Both univariable and multivariable-adjusted logistic regression were used to calculate the odds ratio (OR) with 95% confidence interval (CI) for the relationship between DII and H. pylori infection. The possible nonlinear relationships between DII and the all-cause mortality were further evaluated on a continuous scale with restricted cubic spline (RCS) curves based on the multivariable Cox proportional hazards models and four nodes at the fixed percentiles of 5%, 35%, 65% and 95% of the distribution of DII. To examine the association of DII with all-cause mortality, Cox proportional hazards models were used to calculate hazard ratios (HRs) with 95% CIs. To maximize the available data and limit potential overadjustment for variables that could mediate associations between DII and mortality, three models were estimated: in model 1, age, gender and BMI were adjusted; in model 2, age, gender, BMI, ethnics, education, smoke and drink were adjusted; and in model 3, total cholesterol (TC), creatinine, diabetes mellitus and hypertension were additionally added into model 2. DII scores (as continuous variable) were also categorized into tertiles and then applied in Cox proportional hazards models with tertile 1 as the reference group. The event-free survival rates among the groups were estimated by the Kaplan–Meier method and compared by the log-rank test.

A two-sided p < 0.05 was considered statistically significant. All analyses were performed using SPSS version 26.0 (IBM Corp, Armonk, NY, USA) and R (version 4.2.2) [24–26].

## Results

### Study participants and baseline characteristics

A total of 4, 370 American adults were included finally, of which 1, 891 participants were H. pylori positively infected (Fig. 1; Table 1). The average age was 46.3 ± 20.2 years, and 53.1% were female. The mean DII score was 0.53 ± 1.40. Specifically, the H. pylori positive group had a higher proportion of older individuals, males, individuals with lower socioeconomic status, lower educational attainment, current smokers, former drinkers, individuals with diabetes, hypertension, higher body mass index (BMI), and elevated levels of blood lipids. Furthermore, the H. pylori positive group demonstrated a higher incidence of atherosclerotic cardiovascular disease (ASCVD) and other related conditions.

**Table 1:** Baseline characteristics of participants with different H. pylori infection status

	All (N = 4370)	Hp negative (N = 2479)	Hp positive (N = 1891)	*P* value
Age (years)	46.3 (20.2)	43.5 (20.1)	50.1 (19.6)	< 0.001
Sex				0.028
Female	2319 (53.1%)	1352 (54.5%)	967 (51.1%)	
Male	2051 (46.9%)	1127 (45.5%)	924 (48.9%)	
BMI (kg/m^2^)	28.1 (6.27)	27.9 (6.30)	28.3 (6.22)	0.021
Race				< 0.001
Mexican American	1284 (29.4%)	468 (18.9%)	816 (43.2%)	
Non-Hispanic Black	799 (18.3%)	367 (14.8%)	432 (22.8%)	
Non-Hispanic White	1873 (42.9%)	1444 (58.2%)	429 (22.7%)	
Other Races	414 (9.47%)	200 (8.07%)	214 (11.3%)	
Poverty	2.47 (1.61)	2.79 (1.64)	2.04 (1.46)	< 0.001
Education				< 0.001
College or above	1533 (35.2%)	1111 (44.9%)	422 (22.4%)	
High school or equivalent	1110 (25.5%)	731 (29.6%)	379 (20.2%)	
Less than high school	1714 (39.3%)	634 (25.6%)	1080 (57.4%)	
Smoke				0.033
Former	1043 (26.8%)	580 (26.9%)	463 (26.8%)	
Never	2062 (53.0%)	1175 (54.4%)	887 (51.3%)	
Now	782 (20.1%)	403 (18.7%)	379 (21.9%)	
Alcohol drinking				< 0.001
Never	565 (15.2%)	297 (14.2%)	268 (16.4%)	
Former	765 (20.5%)	369 (17.6%)	396 (24.2%)	
Mild	1189 (31.9%)	725 (34.6%)	464 (28.4%)	
Moderate	504 (13.5%)	318 (15.2%)	186 (11.4%)	
Heavy	705 (18.9%)	385 (18.4%)	320 (19.6%)	
Diabetes mellitus	504 (11.5%)	210 (8.47%)	294 (15.5%)	< 0.001
Hypertension	1622 (37.1%)	818 (33.0%)	804 (42.5%)	< 0.001
SBP (mmHg)	125 (21.0)	123 (19.4)	129 (22.4)	< 0.001
DBP (mmHg)	71.1 (12.6)	70.6 (12.3)	71.8 (13.0)	0.004
Fasting glucose (mg/dL)	103 (35.6)	98.8 (27.2)	108 (43.9)	< 0.001
Fasting insulin (umol/mL)	14.0 (13.1)	13.3 (10.7)	14.8 (15.8)	0.013
HbA1c (%)	5.52 (1.12)	5.37 (0.92)	5.71 (1.30)	< 0.001
Total bilirubin (umol/L)	9.71 (5.04)	9.75 (5.33)	9.66 (4.62)	0.585
Creatinine (mg/dL)	65.6 (49.7)	65.6 (50.2)	65.6 (49.0)	0.985
Fasting triglyceride (mmol/L)	1.64 (1.22)	1.54 (1.04)	1.76 (1.41)	< 0.001
Fasting total cholesterol (mg/dL)	202 (42.5)	200 (42.1)	203 (43.1)	0.044
HDL-C (mg/dL)	51.0 (15.2)	52.0 (15.3)	49.7 (15.0)	< 0.001
LDL-C (mg/dL)	123 (35.5)	122 (35.4)	124 (35.7)	0.268
CRP (mg/dL)	0.50 (0.98)	0.47 (0.99)	0.53 (0.98)	0.039
ASCVD	372 (9.55%)	181 (8.37%)	191 (11.0%)	0.006
Stroke	128 (3.29%)	58 (2.69%)	70 (4.04%)	0.024
Heart attack	165 (4.24%)	79 (3.66%)	86 (4.97%)	0.053
DII	0.53 (1.40)	0.44 (1.41)	0.65 (1.36)	< 0.001

*Hp* Helicobacter Pylori, *SBP* systolic blood pressure, *DBP* diastolic blood pressure, *BMI* body mass index, *HbA1c* glycated hemoglobin, *HDL* high-density lipoprotein, *LDL* low-density lipoprotein, *CRP* C-reactive protein, *ASCVD* atherosclerotic cardiovascular disease, *DII* dietary inflammatory index

### Associations between DII score and H. pylori infection

We performed linear regression analysis to examine the correlation between variables and H. pylori infection in adults, as displayed in Table 2. In the multivariate analysis, demographic characteristics such as sex (P = 0.062) and BMI (P = 0.277) were not associated with H. pylori status, but age, races, education were significantly associated with H. pylori infection. Smoking was associated with H. pylori (P = 0.028), while drinking alcohol was not associated with H. pylori status (P value = 0.516). DII score had a statistically significant association with H. pylori status.

**Table 2:** Risk factors for H. pylori infection in adults in NHANES 1999–2018

Variable	β	Standard error	95%CI	*P* value
Age	0.010	0.001	(0.008 to 0.013)	0.001
Sex	0.070	0.038	(− 0.004 to 0.145)	0.062
BMI	0.003	0.003	(− 0.003 to 0.009)	0.277
Races	0.295	0.018	(0.259 to 0.331)	0.001
Education	− 0.236	0.022	(− 0.280 to 0.192)	0.001
Smoke	0.084	0.038	(0.009 to 0.158)	0.028
Alcohol drink	0.010	0.015	(− 0.020 to 0.040)	0.516
Diabetes mellitus	− 0.004	0.056	(− 0.113 to 0.105)	0.937
Hypertension	− 0.025	0.042	(− 0.108 to 0.058)	0.553
Total cholesterol	–	0	(− 0.001 to 0.001)	0.884
DII	0.031	0.013	(0.005 to 0.057)	0.018

*BMI* body mass index, *DII* dietary inflammatory index

We then conducted a multivariable logistic regression analysis to examine the association between DII and H. pylori infection risk (Table 3). When DII levels were examined as a continuous variable in model 3, for one SD increase in the DII, adjusted odds ratio (OR) for H. pylori infection was 1.08 (95% CI: 1.02–1.14). When DII was assessed as quartiles, in the initial model (model 0), participants in the 2 higher tertiles of DII score (T2, T3) had a significantly higher risk of H. pylori infection compared to those in the lowest tertile (T1), and H. pylori infection was positively correlated with DII score both in T2 and T3 tertiles. The same phenomenon was also observed after adjusting for age, sex and BMI in model 1, and the association was attenuated. Specifically, the risk of H. pylori infection remained significantly higher in the T3 compared to the T1 with an odds ratio (OR) of 1.51 and a 95% confidence interval (CI) of (1.30, 1.77). Further adjustment for races、education、smoking and alcohol drink in model 2 didn’t attenuate the association. In model 3, we additionally adjusted for comorbidities, like total cholesterol, diabetes mellitus and hypertension, significant positive associations were observed. In particular, individuals in the highest tertile of the DII score had the highest risk of H. pylori infection compared to those in the lowest tertile. The odds ratio (OR) for this comparison was 1.30, with a 95% confidence interval (CI) of 1.07–1.57. In addition, the positive correlation between H. pylori infection and DII score in T2 and T3 tertiles also persisted in model 2 and model 3.

**Table 3:** Associations between DII score and H. pylori infection

Variable	Range	Event/total	OR (95%CI)			
			Model 0	Model 1^a^	Model 2^b^	Model 3^c^
Continuous variables						
DII		1891/4370	1.11 (1.07–1.16)***	1.14 (1.09–1.19)***	1.07 (1.01–1.13)*	1.08 (1.02–1.14)**
Categorical variable						
T1 group	− 3.99 to 0.00	560/1455	Ref.	Ref.	Ref.	Ref.
T2 group	0.00 to 1.35	647/1461	1.27 (1.10–1.47)***	1.36 (1.17–1.59)***	1.17 (0.98–1.40)	1.26 (1.05–1.52)**
T3 group	1.35 to 3.99	684/1454	1.42 (1.23–1.65)***	1.51 (1.30–1.77)***	1.25 (1.04–1.51)*	1.30 (1.07–1.57)**

^a^Model 1 adjusted for age, sex, BMI

^b^Model 2 adjusted for age, sex, BMI, races, education, smoke and alcohol drink

^c^Model 3 adjusted for age, gender, BMI, races, education, smoke, alcohol drink, total cholesterol, diabetes mellitus and hypertension

^*^P < *0.05,* **P < *0.01,* ***P < *0.001*

### Correlation between DII and all-cause mortality

**Fig. 2:**
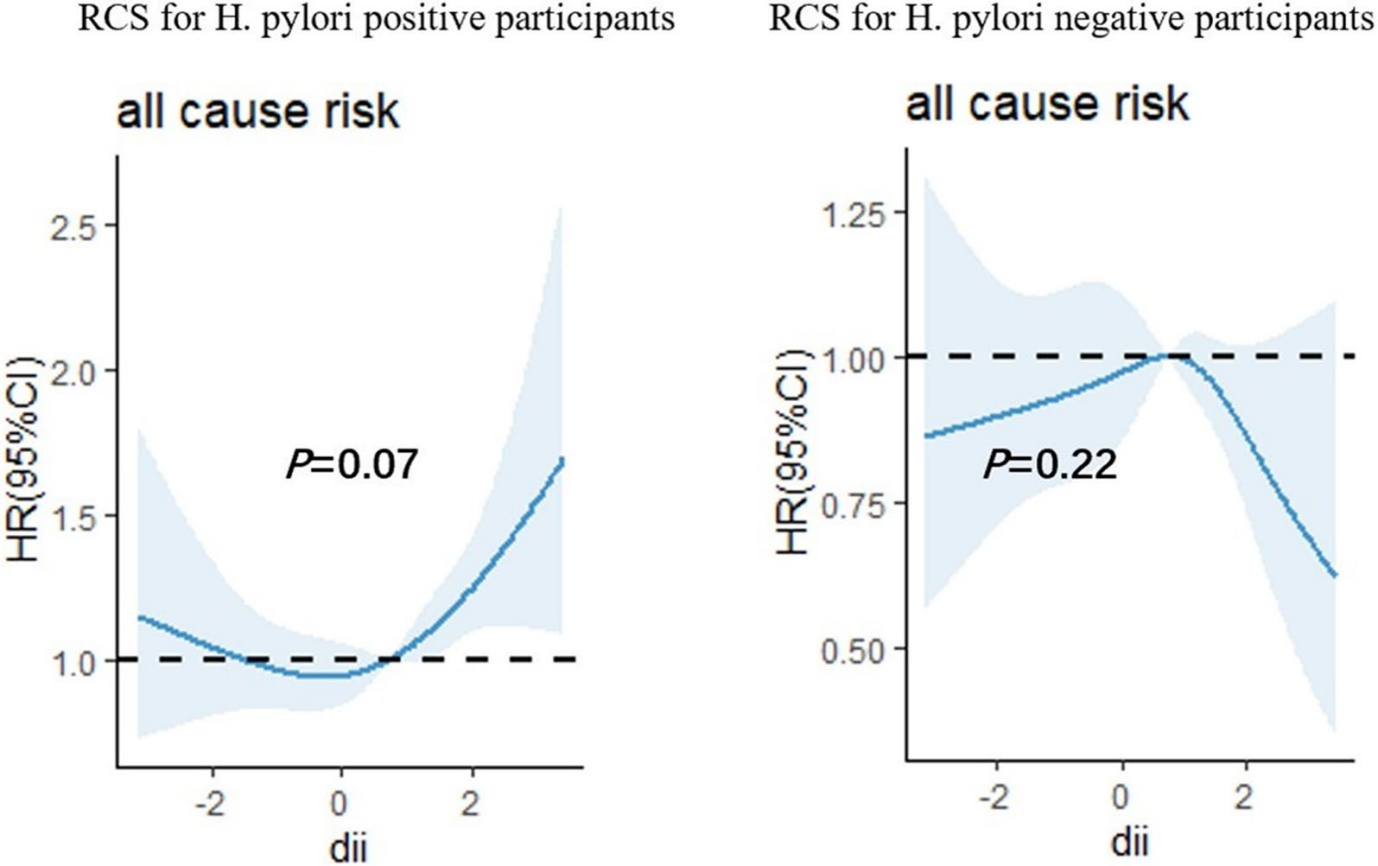
Restricted cubic spline (RCS) for the association between DII and the risks of all-cause death in patients with or without H. pylori infection.

Among the total participants, a total of 1,248 individuals (28.56%) experienced mortality. The relationship between DII and all-cause mortality was further assessed by RCS curves (Fig. 2). The RCS analysis revealed DII, as a continuous variable, was positively associated with an increased adjusted risk of all-cause mortality in H. pylori infected patients (P = 0.07). However, the analysis did indicate that high levels of DII were not associated with an increased risk of death in H. pylori negatively infected population.

**Fig. 3:**
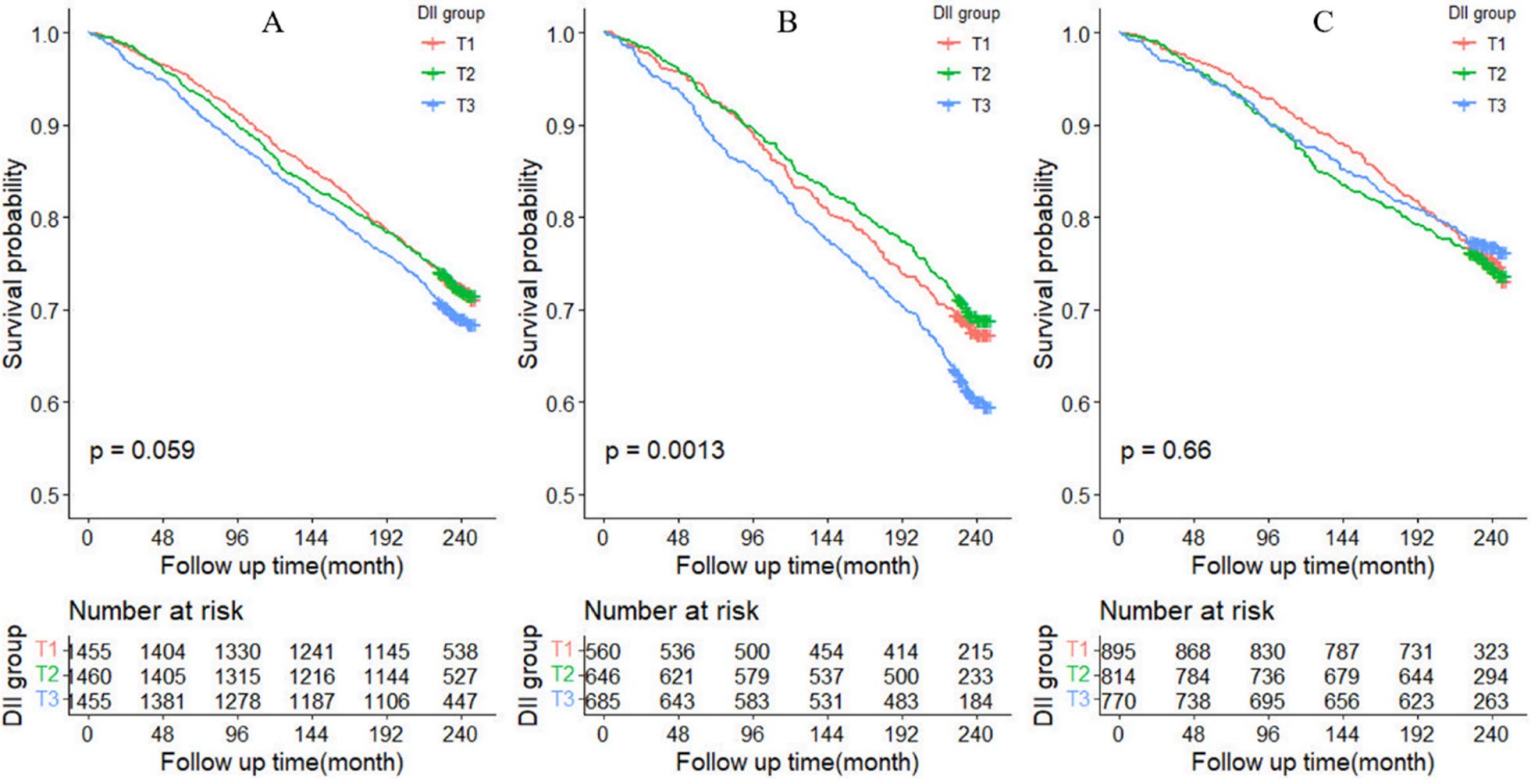
**A** Kaplan–Meier survival estimates for all-cause mortality in all participants. **B** Kaplan–Meier survival estimates for all-cause mortality in H. pylori positively infected participants. **C** Kaplan–Meier survival estimates for all-cause mortality in H. pylori negatively infected participants

The analysis using Kaplan–Meier survival curves demonstrated that higher DII scores were associated with higher all-cause mortality in H. pylori positively infected participants (P-log rank = 0.0013, as depicted in Fig. 3B) while not significantly associated with mortality in all participants or in H. pylori negatively participants (as depicted in Fig. 3A, C). As continuous variable, in model 3, for one SD increase in the DII, adjusted hazard ratio (HR) for mortality was 1.16 (95% CI: 1.08–1.24) in H. pylori positive participants and 1.06 (95% CI: 1.00–1.13) in H. pylori negative population. While DII was assessed as categorical variable, after adjusting potential risk factors, including age, gender, BMI, races, education, smoke, alcohol drink, total cholesterol, diabetes mellitus and hypertension, compared to the T1 group, the T3 group (HR: 1.66, 95% CI: 1.35–2.06; p < 0.001) exhibited a lower risk of death in H. pylori positively infected people (Table 4), which was not found in H. pylori negatively infected participants (Table 5).

**Table 4:** Associations of DII score and all-cause mortality in H. pylori positively infected participants

Variable	Event/total	HR (95%CI)			
		Model 0	Model 1^a^	Model 2^b^	Model 3^c^
Continuous variables					
DII	645/1891	1.08 (1.02–1.14)**	1.16 (1.09–1.23)***	1.15 (1.08–1.23)***	
Categorical variable					
T1 group	180/560	Ref.	Ref.	Ref.	Ref.
T2 group	197/647	0.93 (0.76–1.14)	1.19 (0.97–1.46)	1.19 (0.96–1.47)	1.15 (0.93–1.43)
T3 group	268/684	1.29 (1.06–1.55)**	1.65 (1.36–2.02)***	1.60 (1.30–1.97)***	1.66 (1.35–2.06)***

^a^Model 1 adjusted for age, sex, BMI

^b^Model 2 adjusted for age, sex, BMI, races, education, smoke and alcohol drink

^c^Model 3 adjusted for age, gender, BMI, races, education, smoke, alcohol drink, total cholesterol, diabetes mellitus and hypertension

^*^P < *0.05*, **P < *0.01*, ***P < *0.001*

**Table 5:** Associations of DII score and all-cause mortality in H. pylori negatively infected participants

Variable	Event/total		HR (95%CI)		
		Model 0	Model 1^a^	Model 2^b^	Model 3^c^
Continuous variables					
DII	603/2479	0.99 (0.93–1.04)	1.11 (1.05–1.17)***	1.08 (1.01–1.14) *	1.06 (1.00–1.13)
Categorical variable					
T1 group	220/895	Ref.	Ref.	Ref.	Ref.
T2 group	205/814	1.05 (0.87–1.27)	1.38 (1.14–1.68)***	1.30 (1.06–1.59)**	1.28 (1.05–1.58)*
T3 group	178/770	0.96 (0.78–1.16)	1.26 (1.03–1.55)*	1.12 (0.90–1.40)	1.11 (0.89–1.39)

^a^Model 1 adjusted for age, sex, BMI

^b^Model 2 adjusted for age, sex, BMI, races, education, smoke and alcohol drink

^c^Model 3 adjusted for age, gender, BMI, races, education, smoke, alcohol drink, total cholesterol, diabetes mellitus and hypertension

^*^P < *0.05,* **P < *0.01,* ***P < *0.001*

## Discussion

This study examined the relationship between the DII score and H. pylori infection, and all-cause mortality in patients with or without H. pylori infection, utilizing data from NHANES 1999–2018. Our results demonstrated that subjects with a higher DII score had a higher H. pylori infection risk. Notably, the effect of a pro-inflammatory diet, as indicated by elevated DII scores, on mortality was observed solely among individuals who tested positive for H. pylori infection.

Existing evidence suggests that diet plays a crucial role in the development of H. pylori infection, highlighting the significance of protective dietary factors from a public health perspective. While much of the nutritional research has traditionally focused on individual nutrients or specific foods concerning disease outcomes, considering the overall diet can provide more comprehensive insights since individuals consume a combination of nutrients and foods. Dietary indices, such as the DII, offer a valuable approach in this regard as they capture the overall quality of the diet. Notably, numerous studies have documented a substantial association between the DII and various inflammation-related diseases, including stroke [27], depression [28], and nonalcoholic fatty liver [29]. These findings underscore the potential of assessing the overall diet in understanding the relationship between dietary factors and disease outcomes. Our study outcomes were consistent with prior research demonstrating a consistent inverse association between the diet antioxidant index (DAI), which comprehensively considers the total dietary antioxidant properties, and H. pylori infection [10]. To the best of our knowledge, our study was the first to explore the relationship between the DII and H. pylori infection, contributing valuable insights to the existing body of knowledge.

Additionally, emerging research suggests that the composition of the gastrointestinal microbiota is profoundly influenced by diet, and there is evidence suggesting that DII is linked to the presence of certain microbial species in the gut [30]. Colonization of H. pylori in the stomach leads to alterations in the gastric microbiota and a decrease in bacterial diversity, thereby impacting the gut microbiome of the host [31]. Several studies have observed greater diversity in the gastrointestinal tract ecosystem and linked the presence of H. pylori to variations in the structure of the microbiome [32]. The changes in the gut microbiome induced by initial H. pylori acquisition can also reflect the host's immune status and, consequently, the development of various diseases. This may also account for the positive association between the DII score and H. pylori infection.

According to the survival analysis in our study, higher DII scores were associated with a higher risk of all-cause mortality, especially significant among H. pylori infected participants. These findings align with previous research indicating that high-DII diets can accelerate the rate of telomere shortening, which is associated with an increased risk of mortality in the general population [33]. These diets, characterized by high DII scores, are also associated with elevated levels of inflammatory cytokines such as C-reactive protein, interleukin 6, and tumor necrosis factor-alpha. These inflammatory markers are known to contribute to a higher risk of chronic diseases and mortality [34], including obesity, diabetes, and cardiovascular disease [35]. In fact, a previous meta-analysis has provided evidence supporting the association between a pro-inflammatory diet and increased risks of cardiovascular disease and mortality [36]. On the other hand, A high DII diet has been found to increase the permeability of the gastrointestinal mucosal barrier, which may be associated with changes in tight junctions [37], which are crucial protein structures responsible for maintaining the barrier's integrity. Consequently, substances, including H. pylori, can pass more easily through the barrier and enter the bloodstream. As for H. pylori infection, it has been associated with an increased risk of both cardiovascular disease and gastric cancer, making it a significant contributing factor to mortality. Mohamed Riad has ever found that H. pylori was associated with coronary artery disease through mechanisms such as dyslipidemia, cross-reactivity, hyperhomocysteinemia [38].

## Limitations

Firstly, the use of a Food Frequency Questionnaire (FFQ) for dietary assessment may have introduced inaccuracies and misclassification of dietary intakes. Additionally, participants' reports of past dietary habits may have been affected by recall bias, especially among patients whose lifestyle changes when developing symptomatic H. pylori infection. Secondly, the significant association between DII and H. pylori infection could be influenced by unmeasured or residual confounding factors [39]. Finally, the study lacked data on cooking methods, which could potentially alter the inflammatory content of foods.

## Conclusion

In our sample population, high DII scores are associated with higher risks of H. pylori infection. DII is significantly correlated with all-cause mortality risk in H. pylori positively infected population while not in H. pylori negatively infected participants. These outcomes highlight that the DII holds its own predictive value and offers valuable insights for evaluating the dietary management of individuals with H. pylori infection.

## Data Availability

The raw data supporting the conclusions of this article can be found here: https://www.cdc.gov/nchs/nhanes/.
